# The Use of Massive Endoprostheses for the Treatment of Bone Metastases

**DOI:** 10.1155/2007/62151

**Published:** 2007-07-11

**Authors:** D. H. Park, P. K. Jaiswal, W. Al-Hakim, W. J. S. Aston, R. C. Pollock, J. A. Skinner, S. R. Cannon, T. W. R. Briggs

**Affiliations:** The Royal National Orthopaedic Hospital, Stanmore, Middlesex HA7 4LP, UK

## Abstract

*Purpose*. We report a series of 58 patients with metastatic bone disease treated with resection and endoprosthetic reconstruction over a five-year period at our institution. *Introduction*. The recent advances in adjuvant and neoadjuvant therapy in cancer treatment have resulted in improved prognosis of patients with bone metastases. Most patients who have either an actual or impending pathological fracture should have operative stabilisation or reconstruction. Endoprosthetic reconstructions are indicated in patients with extensive bone loss, failed conventional reconstructions, and selected isolated metastases. *Methods and Results*. We identified all patients who were diagnosed with metastatic disease to bone between 1999 and 2003. One hundred and seventy-one patients were diagnosed with bone metastases. Metastatic breast and renal cancer accounted for 84 lesions (49%). Fifty-eight patients with isolated bone metastasis to the appendicular skeleton had an endoprosthetic reconstruction. There were 28 males and 30 females. Twelve patients had an endoprosthesis in the upper extremity and 46 patients had an endoprosthesis in the lower extremity. The mean age at presentation was 62 years (24 to 88). At the time of writing, 19 patients are still alive, 34 patients have died, and 5 have been lost to follow up. Patients were followed up and evaluated using the musculoskeletal society tumour score (MSTS) and the Toronto extremity salvage score (TESS). The mean MSTS was 73% (57% to 90%) and TESS was 71% (46% to 95%). Mean follow-up was 48.2 months (range 27 to 82 months) and patients died of disease at a mean of 22 months (2 to 51 months) from surgery. Complications included 5 superficial wound infections, 1 aseptic loosening, 4 dislocations, 1 subluxation, and 1 case, where the tibial component of a prosthesis rotated requiring open repositioning. *Conclusions*. We conclude that endoprosthetic replacement for the treatment of isolated bone metastases is a reliable method of limb reconstruction in selected cases. It is associated with low complication and failure rates in our series, and achieves the aims of restoring function, allowing early weight bearing and alleviating pain.

## 1. INTRODUCTION

Bony metastases are the most common neoplasms of bone and the skeleton is the third most common site for metastatic diseases, after the lung and liver [1]. Advances in adjuvant and neoadjuvant therapies, especially in the fields of hormonal therapy and chemotherapy, have improved the prognosis of patients with cancer. This has subsequently led to an increase in the incidence of bony metastases and resultant pathological fractures of the long bones. The management of the patient with a pathological fracture presents a challenge to the orthopaedic surgeon and necessitates a multidisciplinary approach. A consensus statement by the British Orthopaedic Association and the British Orthopaedic Oncology Society has highlighted the fact that there remains a low level of awareness in the hospital and primary-care settings of what can be achieved in the management of metastatic bone diseases [2, 3]. Skeletal complications can have a marked effect on the patient's quality of life, with bone pain being the most frequent clinical symptom. An actual or impending pathological fracture impacts on the patient's function and mobility. In principle, the aims of treatment should be to relieve pain and restore function by stabilising pathological fractures [2, 4–6]. Stabilising impending of actual pathological fractures allows early resumption of ambulation, which significantly improves patients' quality of life [7]. In addition to stabilisation, orthopaedic constructs should allow immediate weight bearing and be designed to last the expected lifetime of the patient [2, 8]. Fracture healing is often poor in diseased or irradiated bone and the surgeon must take into account the fact that these fractures may not unite [9]. Metastatic lesions with extensive bone loss or pathological fractures affecting adjacent joints may be treated with resection and an endoprosthetic reconstruction. The load-bearing characteristics of endoprosthesis offer immediate postoperative stability, and facilitate rapid rehabilitation [10, 11]. Over the last 20 years, the availability and improvement of modular endoprosthesis have improved the treatment of metastatic bone disease, particularly in the treatment of isolated bone metastasis, failed conventional reconstructions and lesions with extensive bone loss [12]. In selected cases, isolated lesions such as a metastasis from renal cell cancer are treated with complete excision and endoprosthetic reconstruction with the intent to cure [4, 13].

The purpose of this paper is to review our experience of patients with metastatic bone disease from carcinoma that had resection and an endoprosthetic reconstruction at our hospital over a five-year period.

## 2. METHODS

We performed a retrospective review of all patients with bone metastases referred to our regional musculoskeletal tumour centre from January 1999 to December 2003. Patients were identified from the tumour database. We determined the patient demographics, indications for treatment and the complications in patients who had resection of metastatic bone lesions and endoprosthetic reconstruction. The following inclusion criteria are applied for the sample collection: (1) a known metastatic lesion in the appendicular skeleton on the basis of histological diagnosis; and (2) no previous resection and endoprosthetic reconstruction. Other information, namely, age, gender, primary lesion if known, site of lesion, and duration of follow-up period, were also noted. All patients referred to our institution are discussed in a multidisciplinary team setting, attended by oncologists, radiologists, orthopaedic surgeons, and other allied health professionals. Decisions regarding prophylactic surgery for patients with impending pathological fractures are made based on Mirels [14] scoring system (Table 1), with a score of >8 necessitating operative stabilisation.

The indications for endoprosthetic reconstruction were isolated single metastases in the long bones, lesions involving adjacent joints, and large lesions with extensive bone loss. In principle, these metastatic lesions were excised in a similar manner to primary bone tumours. Where possible, wide soft tissue margins were obtained and the shaft of the long bone was transected at least 2 cm away from the extent of the disease. In view of the fact that this is palliative surgery, important neurovascular structures were usually preserved at the expense of wide margins in order to maximise functional outcome. In patients with intraarticular spread of tumour, we performed conventional joint replacement surgery and did not attempt extraarticular resections. In the case of proximal femoral, distal femoral and proximal tibial reconstruction modular endoprosthetic tumour system (METS, Stanmore Implants Worldwide Ltd, Stanmore, Middlesex, UK) were used as it became available. For the proximal femoral replacements, this was in 2001 and for the distal femoral and proximal tibial replacements, this was in 2003. These above-mentioned prostheses are modular, off-the-shelf endoprosthetic reconstruction systems. For other tumour locations and before these dates, surgery required the manufacture of custom-made implants (Stanmore Implants Worldwide Ltd).

In patients requiring proximal femoral replacement and whose disease spared the greater trochanter, this structure was osteotomised and reattached to the endoprostheses using a trochanteric reattachment plate and screws. The proximal femoral endoprostheses contain a spiked, hydroxyapatite coated shoulder with two screw holes for this specific purpose. This enables gluteus medius and minimus to be reattached thereby preserving abductor function. Postoperative radiotherapy was offered to all patients with pathological fractures and those whose resection margins were positive. We did not routinely offer radiotherapy to patients who had a successful wide excision.

Functional outcome was assessed using the system adopted by the musculoskeletal tumour Society (MSTS) for the functional evaluation of reconstructive procedures after skeletal resection [15], and a patient-reported measure of disability, the Toronto extremity salvage score (TESS) [16]. The MSTS score is a clinician scored system assessing pain, function, and emotional acceptance in patients for upper and lower extremities. Patients with lower extremity reconstructions were also evaluated with regard to walking ability, gait, and the use of walking aids. Patients with upper extremity reconstructions were evaluated for manual dexterity, hand positioning, and lifting ability. The TESS was developed as a disease-specific measure for patients undergoing limb salvage surgery for tumours of the extremity. It evaluates physical disability based on the patients' report of their function using a self-administered questionnaire, which rates the difficulty experienced in performing certain activities. Both the MSTS and TESS scores are represented as a percentage, with a higher percentage indicating better functional outcome.

## 3. RESULTS

Between January 1999 and December 2003, 171 patients were diagnosed with metastatic bone tumours from carcinoma. Fifty-eight of which underwent an endoprosthetic reconstruction. There were 28 males and 30 females with a mean age at diagnosis of 62 years (range 24 to 88). The most common underlying diagnosis was renal cell carcinoma in 27 (46.6%) of patients, followed by breast carcinoma in 10 (17.2%), unknown primary carcinoma in 8 (13.7%), lung carcinoma in 4, squamous cell carcinoma in 2, prostate carcinoma in 2, thyroid, oesophageal, ovarian, and bladder carcinoma in 1 patient each and a phaeochromocytoma (Table 2). Forty-six patients had lower extremity lesions, which were treated with 31 proximal femoral replacements, 11 distal femoral replacements, and 4 proximal tibial replacements. Twelve had upper extremity lesions, treated with 7 proximal humeral replacements, 4 distal humeral replacements, and 1 humeral diaphyseal replacement.

There were 5 superficial wound infections (8.6%), all of which resolved with oral antibiotics. Four dislocations occurred in the proximal femoral replacements group (12.9%). Three were reduced closed and one required an open reduction without requiring component repositioning and this patient was subsequently managed in an abduction brace for 8 weeks. There was one case of aseptic loosening in the humeral diaphyseal replacement, which required revision, one subluxation in a proximal humeral replacement, and one case of component malposition. The tibial component of a distal femoral replacement was found to be rotated 180 degrees requiring open exploration and repositioning.

Thirty-four patients had died at a mean of 22 months (range 2 to 51 months) from surgery and 5 were lost to follow up. The remaining 19 had a mean follow-up of 48.2 months (range 27 to 82 months). They were functionally evaluated with the MSTS and the TESS scores (Table 3). For the group as a whole, the mean MSTS score was 73% (range from 57 to 90%) and TESS was 71% (range 46 to 95%). Looking specifically at lower limb reconstruction, the mean MSTS score was 77.9% (range 57 to 90%) and the mean TESS was 75.6% (range 46 to 95%). The group with proximal femoral replacements (11 patients) had a mean MSTS score of 72.4% (range 57 to 83) and a mean TESS of 68.4% (range 46 to 84). The group with distal femoral replacements (4 patients) had a mean MSTS score of 75% (range 60 to 90) and a mean TESS of 77.5% (range 63 to 95). One patient with a proximal tibial replacement had an MSTS score of 73% and a TESS score of 72%. In the upper limb, two patients with proximal humeral replacements had MSTS scores and TESS of 72% and 70%, and 73% and 71%, respectively. One patient with a distal humeral replacement had an MSTS score of 76% and a TESS of 77%.

No patients had local recurrence or required subsequent amputation and there was one revision of the humeral diaphyseal replacement as described above.

## 4. DISCUSSION

Endoprosthetic reconstruction has a role in the management of metastatic lesions with extensive bone loss, failure of conventional reconstruction, and large isolated lesions with the aim being curative. Although the conventional treatment of metastatic bone lesions with plates and intramedullary devices supplemented with methylmethacrylate is well established [7], lesions that involve adjacent joints often require resection and reconstruction to allow early and full weight bearing. The purpose of this study was to review our experience with endoprosthetic replacements and to objectively assess patient outcome using both a clinician-reported and a patient-reported score. The majority of the endoprosthetic reconstructions in our series were proximal femoral replacements, a finding reflected in other series of endoprosthetic replacements for bone metastases [5, 12], with the proximal femur being the most common site of long-bone involvement by metastatic disease [8, 17, 18]. The hip joint must bear as much as six times body weight and this necessitates that reconstruction must provide immediate stability and prolonged durability. This strongly favours the use of an endoprosthetic replacement rather than internal fixation [8]. Conventional fixation of pathological fractures or large lytic lesions especially around the hip or proximal femur has a high failure rate when compared to a standard or tumour prosthetic replacement [5, 19, 20]. It is therefore our preferred method of treatment to carry out a resection and endoprosthetic replacement for large lesions of the proximal femur. The extent of tumour in the proximal femur dictated the method of abductor repair and if the trochanter could be spared with an adequate margin of bone between the tumour and a trochanteric osteotomy, then the trochanter was reattached in the manner described previously. Tumour involving the trochanter resulted in resection of the proximal femur including the trochanter and a soft tissue abductor repair was done. In our series of 11 patients, only one patient was suitable for a trochanteric osteotomy and reattachment to the prosthesis. This patient subsequently had a dislocation on her first postoperative day and underwent a closed reduction. At 31 months follow-up, her MSTS score was 57% and her TESS was 46%. Due to the small numbers we are unable to comment on whether trochanteric reattachment significantly affects functional outcome or hip abductor function. Of the 11 patients with proximal femoral lesions, seven presented with a pathological fracture. Patients who had a pathological fracture on presentation had a mean MSTS score of 69.1% (range 57 to 80%) and a mean TESS of 65.9% (46 to 82%). Patients who presented without a pathological fracture had a mean MSTS score of 78% (range 70 to 83%) and a mean TESS of 72.8% (range 53 to 84%). The difference in scores are not statistically significant and larger studies will be required to determine if patients who present with pathological fractures have poorer functional outcome scores compared to patients who do not. With regard to overall functional outcome, patients with proximal femoral replacements had a mean MSTS score of 72.3% (range 57 to 83%) and a mean TESS score of 68.4% (range 46 to 84%). These scores compare to those reported in other series of proximal femoral endoprosthetic replacements using modular endoprostheses [21, 22].

Table 3 shows the functional outcomes of all the patients who survived 2 years or more who were treated with an endoprosthetic replacement. The functional outcomes for patients with upper and lower limb reconstructions are comparable; however, the number of patients followed up with upper limb reconstructions (three) is too small for any further significant conclusions.

There were five cases of superficial infection which resolved with oral antibiotics alone, but no cases of deep infection. In 58 endoprosthetic replacements, only one patient required a revision for aseptic loosening of a humeral diaphyseal replacement. There were four dislocations in the group of patients who had proximal femoral replacements (31 patients). All dislocations occurred within the first 3 weeks of surgery. Three were reduced closed and one required an open reduction without the need for component repositioning. They were all rehabilitated postreduction in an abduction brace for 8 to 10 weeks. None of these patients experienced any further dislocations. Two of these patients survived more than 2 years and in the latest follow-up, they were mobilising with one walking stick. Functionally their MSTS scores and TESS were 57% and 46%, and 70% and 53%, respectively.

The mean time to death of the 58 patients was 22 months (range 2 to 51 months). This wide range highlights the need for a stable reconstruction that allows early weight bearing, has a low incidence of failure, and outlasts the expected lifetime of the patient.

Our series was associated with relatively few, easily manageable complications and there were no implant failures.

Massive endoprostheses were originally developed for the treatment of primary malignant bone tumours. They have traditionally been custom-designed and hence there was a time delay to manufacture the implant. The gradual introduction of modular endoprostheses has provided greater flexibility making these reconstructions possible, and in shorter time frames, therefore aiding the overall management of metastatic bone disease. According to British Orthopaedic Association guidelines [2], patients should undergo a single procedure that allows early full weight bearing and lasts the expected lifespan of the patient. In our experience the use of an endoprostheses allows these criteria to be met.

Appropriate and prompt surgical management of metastatic bone lesions may be more cost-effective in terms of the overall management of cancer patients. This is reflected in earlier mobilisation and therefore potentially less time spent in hospital. Other studies are needed to assess the impact of these cost savings on hospital, nursing, and community cancer services. Patients who had an endoprosthetic reconstruction in our series were able to return to a good level of function. Careful patient selection is crucial and the surgeon must take into consideration the patient's prognosis, comorbidities, and their ability to participate in postoperative rehabilitation.

## Figures and Tables

**Table 1:** Mirels' Scoring System.

Variable	Score		
	1	2	3

Site	Upper limb	Lower limb	Peritrochanter
Pain	Mild	Moderate	Functional
Lesion	Blastic	Mixed	Lytic
Size*	<1/3	1/3-2/3	>2/3

*As seen on plain X-ray, minimum destruction of cortex in any view. Maximum possible score is 12. If lesion scores 8 or above, then prophylactic fixation is recommended prior to surgery.

**Table 2:** Primary diagnosis of patients undergoing endoprosthetic reconstruction.

Primary diagnosis	Number (%)

Renal carcinoma	27 (46.6)
Breast carcinoma	10 (17.2)
Unknown primary	8 (13.8)
Lung carcinoma	4 (6.9)
Squamous cell carcinoma	2 (3.4)
Prostate carcinoma	2 (3.4)
Thyroid carcinoma	1 (1.7)
Oesophageal carcinoma	1 (1.7)
Phaeochromocytoma	1 (1.7)
Ovarian carcinoma	1 (1.7)
Bladder carcinoma	1 (1.7)

**Table 3:** Functional outcomes of the 19 patients (out of 58) surviving 2 years or more.

Patient	Primary	Age	Operation*	TESS	MSTS	Follow-up (months)

1	Phaeochromocytoma	24	PFR	82	80	39
2	Thyroid carcinoma	42	PFR	72	74	41
3	Oesophageal carcinoma	42	PFR	69	63	35
4	Ovarian carcinoma	45	PFR	65	77	33
5	Breast carcinoma	49	PFR	46	57	31
6	Renal carcinoma	67	PFR	56	63	31
7	Unknown primary	77	PFR	71	70	44
8	Unknown primary	58	PFR	74	78	44
9	Breast carcinoma	68	PFR	53	70	27
10	Breast carcinoma	42	PFR	84	83	58
11	Renal carcinoma	36	PFR	80	81	46
12	Squamous cell carcinoma	49	PTR	72	73	28
13	Renal carcinoma	46	DFR	72	71	82
14	Breast carcinoma	50	DFR	95	90	35
15	Renal carcinoma	56	DFR	80	79	66
16	Renal carcinoma	61	DFR	63	60	73
17	Renal carcinoma	52	PHR	71	73	73
18	Renal carcinoma	54	PHR	70	72	60
19	Unknown primary	59	DHR	77	76	60

*PFR = proximal femoral replacement, PHR = proximal humeral replacement, PTR = proximal tibial replacement, DFR = distal femoral replacement, DHR = distal humeral replacement.
